# The estimated prevalence of exposure to asthmagens in the Australian workforce, 2014

**DOI:** 10.1186/s12890-016-0212-6

**Published:** 2016-04-09

**Authors:** Lin Fritschi, Julie Crewe, Ellie Darcey, Alison Reid, Deborah C. Glass, Geza P. Benke, Tim Driscoll, Susan Peters, Si Si, Michael J. Abramson, Renee N. Carey

**Affiliations:** School of Public Health, Curtin University, GPO Box U1987, Perth, Western Australia 6845 Australia; Monash Centre for Occupational and Environmental Health, School of Public Health & Preventive Medicine, Monash University, Melbourne, Victoria Australia; Sydney School of Public Health, University of Sydney, New South Wales, Australia; School of Population Health, University of Western Australia, Perth, Western Australia Australia

**Keywords:** Occupational asthma, Surveillance, Workplace exposure

## Abstract

**Background:**

There is very little information available on a national level as to the number of people exposed to specific asthmagens in workplaces.

**Methods:**

We conducted a national telephone survey in Australia to investigate the prevalence of current occupational exposure to 277 asthmagens, assembled into 27 groups. Demographic and current job information were obtained. A web-based tool, OccIDEAS, was used to collect job task information and assign exposure to each asthmagen group.

**Results:**

In the Australian Workplace Exposure Study – Asthma (AWES- Asthma) we interviewed 4878 participants (2441 male and 2437 female). Exposure to at least one asthmagen was more common among men (47 %) than women (40 %). Extrapolated to the Australian population, approximately 2.8 million men and 1.7 million women were estimated to be exposed. Among men, the most common exposures were bioaerosols (29 %) and metals (27 %), whilst the most common exposures among women were latex (25 %) and industrial cleaning and sterilising agents (20 %).

**Conclusions:**

This study provides information about the prevalence of exposure to asthmagens in Australian workplaces which will be useful in setting priorities for control and prevention of occupational asthma.

**Electronic supplementary material:**

The online version of this article (doi:10.1186/s12890-016-0212-6) contains supplementary material, which is available to authorized users.

## Background

Work-related (or occupational) asthma is caused by exposure to agents in an occupational setting and includes both work-aggravated asthma (in which workplace exposures provoke or exacerbate symptoms of pre-existing asthma) and newly-occurring occupational asthma [1]. Occupational asthma is divided into two groups: allergic and non-allergic [2]. Allergic occupational asthma is caused by exposures to agents which sensitize the airways. The airways then react to subsequent additional exposure to that agent and may also react to other agents and triggers. Non-allergic occupational asthma is less common (perhaps 10 % of all occupational asthma) and is a result of exposure to high levels of a respiratory irritant (such as hydrogen chloride, ammonia, chlorine etc).

A review of international studies estimated that about 16–17 % of adult onset asthma was caused by occupational exposures [3]. Work-related asthma is one of the few preventable types of asthma. For example, a study in the aluminium industry showed that the incidence of occupational asthma in seven smelters had declined from 9.46/1000 per year in 1992 to 0.36/1000 per year in 2006; a 96.2 % reduction [4]. This reduction was ascribed to improvements in control of exposures, respiratory protection and pre-placement medical assessments.

Several hundred workplace agents have been found to cause occupational asthma, including organic dusts (e.g. wood, flour, animal dander), and chemicals such as isocyanates and glutaraldehyde [5]. Exposure to asthmagens may occur in many occupations including farming, painting, food preparation, nursing and laboratory work [6]. In Finland, it was estimated in 1992 that about 13 % of the workforce were exposed to allergens at work and 30 % were exposed to respiratory contaminants [7]. In the European Community Respiratory Health Survey, about half of the sample reported exposure to “vapors, gas, dust or fumes” in their current job [8]. Similarly, about two thirds of a Norwegian cohort were assessed by a job exposure matrix as being exposed at some time during their working life to at least one of the following agents: biological dust, mineral dust, and gas and fumes [9]. None of these studies identified specific asthmagens and not all dusts are asthmagenic. In New Zealand, 55 % of the population self-reported exposure to at least one group of asthmagens [10]. There are currently no estimates of the number of people exposed to specific asthmagens in workplaces in Australia.

We recently developed a comprehensive and inclusive list of asthmagens relevant for Australian workplaces [11]. It contained 277 asthmagens (in 27 groups) (Table 3) which had been identified from a number of published sources and were all deemed to meet three criteria: (1) there was evidence that the agent was an asthmagen; (2) the agent was used in occupational circumstances; and (3) it was potentially present in Australian workplaces. We subsequently undertook a national cross-sectional survey (The Australian Workplace Exposures Study – Asthma or AWES-Asthma) in 2014 to investigate the prevalence of occupational exposures to these asthmagens. This paper reports the methods and results of this survey.

## Methods

### Subject selection

The survey sample list was obtained from a commercial survey sampling company and included both landline and mobile telephone numbers, along with postal addresses and postcodes. We requested a randomized sample, stratified to be broadly proportional to the 2011 distribution of the Australian workforce by state and territory [12]. Unfortunately, we discovered after the data collection was complete that the sample provided was not random (56 % of the last names selected began with L and 25 % began with M). The effect of this on our results is not known but is not likely to be substantial.

Each number was telephoned and asked if there was anyone on that number aged 18 to 64 years and currently in paid employment (having worked for one hour or more for pay in the previous week [13]). Exclusion criteria were if the respondent did not have a sufficiently good understanding of spoken English, had a hearing disability or was too ill to complete the telephone survey. Due to the reluctance of male respondents to contribute to research surveys [14] and our previous experience [15] we used a modified interview request for landlines. That is, if there were both male and female eligible workers in a household, the interviewer would request a male in 6 out of 7 calls.

### Data collection

All data were collected by experienced telephone survey staff. Each interview was designed to be completed in less than 15 min. Phone calls were made between 9 am and 8 pm on weekday evenings and between 10 am and 5 pm on weekends.

The interviewers recorded demographic information including age, gender, country of birth, year of arrival in Australia, highest education level achieved, personal and co-workers’ smoking status, business size and whether the respondent was aware of dust or gas monitoring in their work spaces. From the respondent’s residential postcode, we derived the socio-economic indexes for areas (SEIFA) disadvantage score [16] and the accessibility/remoteness index of Australia (ARIA+) score [17].

Preliminary information on the main job for each person was collected to establish whether the respondent’s occupation corresponded to one of the categories of employment that were predetermined by occupational hygienists to be unlikely to result in exposure to any of the 277 asthmagens. These were: office and clerical workers, data processors, flight attendants or pilots, retail sales workers other than food outlets, customer service workers, bank or postal service staff, and correctional services officers. The respondents who were employed in any one of these categories were classified as unexposed and the interview ended at that point.

For the remaining workers, additional information regarding their current job was obtained, including job title, industry type, number of hours worked per week and weeks worked per year. Using this information about a person’s job and industry type, interviewers assigned the respondents to one of 52 job specific modules (JSMs) within the web-based tool OccIDEAS [18]. The JSMs were individually developed for specific jobs where it was considered that exposure to any of the asthmagens could occur and which were reasonably common in Australia. Where a job did not fit any of the 52 JSMs, a Generic JSM was assigned to collect information about tasks commonly carried out.

### Exposure assessments

Each JSM within OccIDEAS contained questions relating to specific tasks which had been identified as determinants of exposure to one or more of the asthmagen groups based on published literature, material data sheets and expert knowledge. Algorithms based on literature and expert opinion were used to assign the likelihood of exposure to each of the listed asthmagen groups (either ‘no’, ‘possible’ or ‘probable’). All automatic assessments were reviewed by project staff and rules were changed where necessary and appropriate. Any such changes were then applied to all assessments using the revised rule. The reviewers also used all available information to categorize the “possible” exposures into either probable or no exposure. Where the information was inadequate to be confident of exposure, we classified the remaining 873 possible assessments as unexposed (0.7 % of all assessments).

### Statistical analysis

Each of the respondents’ job titles were coded using the Australian and New Zealand Standard Classification of Occupations (ANZSCO) [19]. These codes were then classified into 24 occupational groups (Table 1), containing occupations which were broadly similar with regard to exposure to the asthmagen groups.

**Table 1:** Comparisons between the AWES-Asthma sample and the Australian workforce [23] by gender

	Males					Females				
Demographic Characteristic	AWES Sample		Australian Census		*p*-value	AWES Sample		Australian Census		*p*-value
	n	%	n	%		n	%	n	%	
Total	2 441		5 040 849			2 437		4 441 578		
Age					<0.001					<0.001
18–34 years	301	12 %	1 844 844	37 %		227	9 %	1 634 880	37 %	
35–50 years	1 050	43 %	1 957 490	39 %		1 115	46 %	1 751 048	39 %	
51–64 years	1 090	45 %	1 238 515	25 %		1 095	45 %	1 055 650	24 %	
State					1.0					1.0
New South Wales	767	31 %	1 573 658	31 %		760	31 %	1 388 132	31 %	
Victoria	595	24 %	1 272 872	25 %		637	26 %	1 121 241	25 %	
Queensland	471	19 %	1 012 186	20 %		481	20 %	901 345	20 %	
South Australia	166	7 %	367 283	7 %		180	7 %	328 939	7 %	
Western Australia	308	13 %	563 640	11 %		259	11 %	469 323	11 %	
Tasmania	61	2 %	105 692	2 %		46	2 %	98 326	2 %	
Australian Capital Territory	47	2 %	95 380	2 %		44	2 %	90 729	2 %	
Northern Territory	26	1 %	50 138	1 %		30	1 %	43 543	1 %	
Country of Birth					0.06					0.10
Australia	1 913	78 %	3 529 539	70 %		1 937	79 %	3 178 745	72 %	
Other	524	21 %	1 511 310	30 %		496	20 %	1 262 833	28 %	
Education					0.12					0.12
High school or lower	962	39 %	1 949 397	39 %		806	33 %	1 822 204	41 %	
Vocational/Trade	720	29 %	1 904 288	38 %		684	28 %	1 269 804	29 %	
Bachelor or higher	758	31 %	1 187 164	24 %		946	39 %	1 349 570	30 %	
Socioeconomic status					0.57					0.32
Highest Quintile (Most advantaged)	560	23 %	1 403 088	28 %		519	21 %	1 288 370	29 %	
Fourth	483	20 %	1 146 277	23 %		515	21 %	1 018 968	23 %	
Third	519	21 %	1 026 527	21 %		523	21 %	900 151	20 %	
Second	522	21 %	785 385	16 %		504	21 %	683 200	16 %	
Lowest (Least advantaged)	348	14 %	626 538	13 %		374	15 %	509 704	12 %	
Remoteness					<0.001					<0.001
Major City	1 232	50 %	3 617 002	72 %		1 216	50 %	3 207 391	72 %	
Inner regional	933	38 %	858 019	17 %		945	39 %	766 516	17 %	
Outer regional/Remote/Very remote	276	11 %	556 727	11 %		276	11 %	462 011	10 %	
Occupation Group					0.66					0.46
Allied health	11	0 %	56 186	1 %		34	1 %	142 242	3 %	
Carers	16	1 %	50 425	1 %		108	4 %	287 442	7 %	
Cleaning	36	1 %	67 601	1 %		71	3 %	112 962	3 %	
Construction	185	8 %	481 448	10 %		1	0 %	23 653	1 %	
Education	88	4 %	137 470	3 %		343	14 %	364 921	9 %	
Electric/electronic	75	3 %	210 123	5 %		0	0 %	7 064	0 %	
Farming/Animal Worker	178	7 %	142 114	3 %		60	2 %	68 626	2 %	
Food preparation	79	3 %	169 586	4 %		71	3 %	115 877	3 %	
Food Service	18	1 %	90 777	2 %		42	2 %	159 860	4 %	
Gardening	62	3 %	113 707	2 %		17	1 %	18 715	0 %	
Hairdressers	2	0 %	10 931	0 %		22	1 %	70 677	2 %	
Manager-Administration	871	36 %	1 123 391	24 %	0.01	1 129	46 %	1 654 232	39 %	0.15
Manufacturing	64	3 %	155 192	3 %		22	1 %	72 262	2 %	
Mechanical Workers	64	3 %	111 689	2 %		1	0 %	1 681	0 %	
Metal Workers	82	3 %	174 438	4 %		2	0 %	2 574	0 %	
Mining	27	1 %	67 135	1 %		0	0 %	6 181	0 %	
Nurse/Medical	45	2 %	81 407	2 %		227	9 %	290 228	7 %	
Other	23	1 %	151 785	3 %		11	0 %	96 719	2 %	
Painting/Printing	45	2 %	102 195	2 %		4	0 %	23 348	1 %	
Retail	143	6 %	485 707	10 %		205	8 %	585 503	14 %	
Security/safety	48	2 %	115 691	2 %		7	0 %	26 264	1 %	
Technical/engineering	37	2 %	87 799	2 %		40	2 %	62 513	1 %	
Transport	166	7 %	339 713	7 %		18	1 %	36 282	1 %	
Wood workers	76	3 %	135 873	3 %		2	0 %	2 816	0 %	

All analyses were conducted using Stata v14 [20]. Prevalence of any exposure was defined as the proportion of respondents assessed as being probably exposed to at least one of the listed asthmagens in their current job. A dichotomous measure of exposure was used. Odds Ratios (ORs) and 95 % confidence intervals (CIs) were estimated using logistic regression to determine which, if any, demographic factors were associated with any exposure. For the continuous variable age we categorized the data using cut-offs of 35 and 50 based on the methods of Abdolell [21].

We stratified all analyses by gender due to the different profiles of occupation and exposure between genders. Where there was a statistically significant difference between our sample and the labour force, we used raked-weighting [22] to adjust the survey data such that the data structure was made similar to the national labour force population structure [23] in terms of sociodemographic indicators (age group and remoteness for both genders and administrative job (yes/no) for women).

As a visual demonstration of the patterns of exposure among the occupational groups, we calculated Euclidean distances. To do this we compared the differences between prevalences in groups and ranked the groups according to the size of those differences. First we compared the prevalence of all exposures combined in each occupational group with the prevalence of all exposures combined in the manager/administration group (which had low prevalence of exposure). Next we examined each occupational group in turn and compared the prevalence of each exposure group with the prevalence of the asthmagen group “Drugs” (which had low prevalence in all occupational groups). Euclidean distances are used to group similar ranked cells on both axes. In the upper right corner, occupational and asthmagen groups with the highest prevalence are clustered together.

### Ethics, consent and permissions

This study was approved by the Curtin University Human Research Ethics Committee. Informed consent by all participants was presumed by the approval to proceed with the survey questions after a description of the study had been provided.

## Results

**Fig. 1:**
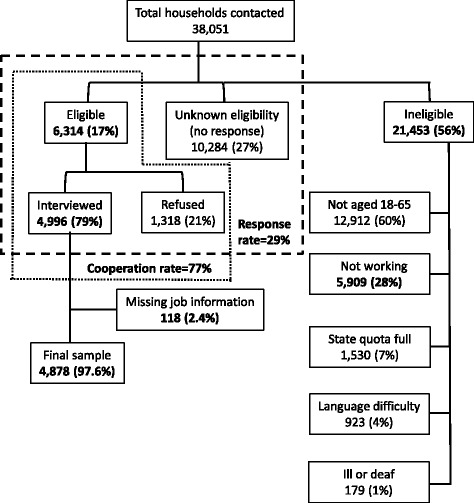
Flow chart of responses to telephone survey cohort

A total of 38,051 telephone numbers were called over the 6 month period of the survey. No response was recorded after 10 call attempts from 10,284 households (“unknown households”), 21,429 contacts were deemed ineligible and 1318 refused to participate (Fig. 1). Job information was incomplete for 118 people who were interviewed. Complete interviews were obtained from 4878 workers (2441 males and 2437 females) resulting in a response fraction (completed interviews/eligible and unknown households) of 29 % and a cooperation fraction (completed interviews/eligible households) of 77 %.

The study population was similar to the Australian working population in terms of state of residence, country of birth (Australia or other), education and socioeconomic status of residence (Table 1). Our sample under-represented younger workers (18–34 years), and those living in major cities. Among occupational groups, our sample over-represented manager/administration workers among males.

Amongst males, in the unadjusted analyses, exposure was more common amongst those aged less than 35 years, those born in Australia, those with a vocational qualification or no post-secondary qualifications, those living in Victoria, those in the lowest SEIFA residential quintiles and those living outside major cities (Table 2). When we adjusted for demographic characteristics and occupational groups, exposure to at least one asthmagen was significantly more common among men with a vocational qualification and those living in Victoria or Tasmania. Among females, after adjustment, only living in an outer regional/remote or very remote area was statistically associated with a higher prevalence of exposure to at least one asthmagen (Table 2).

**Table 2:** Odds ratios (OR) and 95 % confidence intervals (CI) for association between demographic characteristics and probable exposure

	Males			Females		
	Exposed (%)	Unadjusted OR (CI)	Adjusted OR (CI)^a^	Exposed (%)	Unadjusted OR (CI)	Adjusted OR (CI)^a^
Age (years) Linear		**0.99(0.98,0.999)**			1.01(0.99,1.02)	
Age (men)						
18–34 years	53.2	**1.30(1.03,1.68)**	1.32(0.85,2.04)			
35–64 years	46.3	1	1			
Age (women)						
18–50 years				37.9	1	1
51–64 years				43.3	**1.25(1.06,1.47)**	1.06(0.77,1.46)
Country of birth						
Australia	49.2	1	1	41.5	1	1
other	39.9	**0.68(0.56,0.83)**	1.40(0.97,2.03)	36.1	**0.80(0.65,0.98)**	0.87(0.58,1.32)
Highest education level						
Bachelor or higher	28.5	1	1	41.6	1	1
Vocational/trade/TAFE	59.2	**3.64(2.93,4.52)**	**1.90(1.24,2.92)**	40.8	0.96(0.79,1.18)	1.30(0.86,1.96)
High school or lower	53.6	**2.86(2.33,3.50)**	1.34(0.88,2.05)	38.4	0.87(0.72,1.06)	1.13(0.71,1.78)
refused	18.2	0.56(0.12,2.60)	**0.12(0.02,0.91)**	50.0	1.40(0.40,4.87)	2.55(0.31,20.97)
State of residence						
New South Wales	41.7	1	1	35.9	1	1
Victoria	61.3	**2.22(1.78,2.76)**	**1.84(1.19,2.85)**	49.1	**1.72(1.39,2.14)**	0.76(0.47,1.25)
Queensland	45.4	1.18(0.43,3.22)	1.03(0.69,1.53)	37.4	1.06(0.39,2.90)	0.94(0.59,1.50)
Western Australia	40.3	0.94(0.72,1.23)	0.78(0.49,1.23)	40.2	1.20(0.90,1.60)	0.45(0.53,1.67)
South Australia	40.4	0.99(0.92,1.06)	1.49(0.81,2.74)	39.4	1.03(0.96,1.10)	0.97(0.52,1.84)
Tasmania	49.2	1.35(0.80,1.28)	**3.26(1.27,8.36)**	34.8	0.95(0.51,1.78)	0.33(0.08,1.31)
Australian Capital Territory	44.7	1.13(0.62,2.04)	2.07(0.65,6.60)	38.6	1.12(0.60,2.10)	1.44(0.45,4.59)
Northern Territory	38.5	0.87(0.39,1.95)	0.33(0.09,1.16)	33.3	0.89(0.41,1.93)	1.72(0.41,7.16)
SES of residential area						
Highest quintile^b^	38.2	1	1	33.3	1	1
Fourth	41.8	1.16(0.91,1.49)	0.86(0.55,1.34)	38.6	1.26(0.98,1.62)	1.17(0.72,1.91)
Third	51.4	**1.71(1.34,2.18)**	1.02(0.66,1.58)	37.9	1.22(0.95,1.57)	1.00(0.61,1.67)
Second	51.3	**1.72(1.35,2.19)**	0.72(0.45,1.14)	46.2	**1.72(1.34,2.21)**	1.24(0.74,2.08)
Lowest	54.6	**1.94(1.48,2.55)**	0.66(0.40,1.09)	48.4	**1.88(1.43,2.46)**	1.66(0.93,2.95)
unknown	100			0		
Remoteness						
Major cities	37.3	1	1	34.6	1	1
Inner regional	55.1	**2.06(1.73,2.45)**	1.04(0.70,1.53)	45.3	**1.56(1.31,1.86)**	1.23(0.78,1.92)
Outer regional/Remote/very remote	64.1	**3.00(2.29,3.94)**	1.47(0.84,2.56)	48.9	**1.81(1.39,2.35)**	**2.48(1.30,4.72)**

^a^ Adjusted for occupational group and all other demographic characteristics

^b^ SES – socioeconomic status (2 quintiles calculated from deciles of Areas Index of Relative Socio-economic Disadvantage [ABS])

Bold denotes statistically significant differences

As well as the above analysis of exposure to any asthmagen group, we also examined the patterns of exposure to individual agents. Exposure to one or more asthmagens was more common among males (47 %) than females (40 %). When extrapolated to the Australian population, about 4.4 million people (2.8 million males and 1.7 million females) were estimated to be exposed to one or more asthmagen at work. Amongst occupational groups with more than 50 male respondents, exposure to at least one of the asthmagens was most common among farmers and animal workers (97 % exposed), metal workers (96 %), wood workers (96 %), food preparation workers (92 %) and mechanical workers (92 %). Amongst females, the occupational groups with the highest prevalence of exposure were farmers and animal workers (100 %), carers (99 %), cleaners (96 %), food preparation workers (96 %) and nurses (92 %).

The most common asthmagen exposures among males (Table 3) were bioaerosols (29 % of the Australian workforce), metals (27 %), arthropods/mites (25 %), and latex (22 %). Among females (Table 4) the most common exposures were latex (25 %), industrial cleaning and sterilizing agents (20 %), bioaerosols (18 %) and arthropods/mites (16 %). Exposure to isocyanates, which are well-recognized asthmagens, was relatively rare (4 % among men and <1 % among women).

**Table 3:** Prevalence of probable exposure to each asthmagen group in the AWES-Asthma sample and approximate prevalence of exposure in the male Australian working population. CI confidence interval

	Sample		Extrapolated to Australian working population^a^		
Asthmagen group	n	%	n	%	CI
Any asthmagen	1 151	47.2	2 781 500	55	52 to 58
Bioaerosols	633	25.9	1 483 000	29	27 to 32
Metals	563	23.1	1 358 500	27	25 to 30
Arthropods or mites	518	21.2	1 234 500	24	22 to 27
Latex	451	18.5	1 115 500	22	20 to 25
Aldehydes	364	14.9	829 700	16	14 to 19
Industrial cleaning and sterilising agents	264	10.8	683 000	14	12 to 16
Derived from animals	328	13.4	655 800	13	11 to 15
Ammonia	309	12.7	564 600	11	10 to 13
Acrylates	206	8.4	526 800	10	9 to 12
Epoxy	174	7.1	486 400	10	8 to 12
Anhydrides	191	7.8	433 500	9	7 to 10
Other Reactive Chemicals	142	5.8	366 500	7	6 to 9
Foods	121	5.0	359 900	7	6 to 9
Biological Enzymes	136	5.6	357 400	7	6 to 9
Isocyanates	106	4.3	283 300	6	4 to 7
Derived from Plants-Other	188	7.7	270 200	5	4 to 7
Flour	74	3.0	239 400	5	4 to 6
Acids	96	3.9	218 300	4	3 to 6
Soldering	75	3.1	214 700	4	3 to 6
Wood Dusts	86	3.5	200 100	4	3 to 5
Amines	47	1.9	123 500	2	2 to 4
Derived from fish/shellfish	36	1.5	113 400	2	2 to 3
Pesticides	59	2.4	112 400	2	2 to 3
Flowers	23	0.9	36 800	<1	
Ethylene Oxide	14	0.6	34 300	<1	
Drugs	7	0.3	20 200	<1	
Reactive dyes	4	0.2	45	<1	

^a^ Using age, remoteness and manager status for raked weighting

**Table 4:** Prevalence of probable exposure to each asthmagen group the AWES-Asthma sample and approximate prevalence of exposure in the female Australian working population. CI confidence interval

	Sample		Extrapolated to the Australian working population^a^		
Asthmagen group	n	%	n	%	CI
Any asthmagen	984	40.4	1 656 300	37	34 to 40
Latex	601	24.7	980 700	22	20 to 25
Industrial cleaning and sterilising agents	491	20.2	823 500	19	16 to 21
Bioaerosols	439	18.0	728 900	16	14 to 19
Arthropods or mites	398	16.3	676 900	15	13 to 18
Biological Enzymes	279	11.5	467 300	11	9 to 12
Foods	247	10.1	445 900	10	8 to 12
Ammonia	252	10.3	373 500	8	7 to 10
Flour	212	8.7	358 900	8	7 to 10
Aldehydes	201	8.3	307 800	7	6 to 8
Derived from animals	218	9.0	282 900	6	5 to 8
Metals	153	6.3	220 700	5	4 to 6
Flowers	85	3.5	147 500	3	2 to 5
Acrylates	65	2.7	125 300	3	2 to 4
Derived from fish/shellfish	64	2.6	105 300	2	2 to 3
Pesticides	51	2.1	75 500	2	1 to 3
Derived from Plants-Other	55	2.3	62 600	1	1 to 2
Acids	38	1.6	53 700	1	1 to 2
Amines	21	0.9	51 100	1	1 to 2
Drugs	7	0.3	24 400	<1	
Epoxy	19	0.8	22 700	<1	
Isocyanates	11	0.5	11 500	<1	
Other Reactive Chemicals	5	0.2	7 600	<1	
Reactive dyes	4	0.2	36	<1	
Ethylene Oxide	4	0.2	36	<1	
Anhydrides	2	0.1	13	<1	
Soldering	1	0.0	4	<1	
Wood Dusts	3	0.1	1	<1	

^a^ Using age, remoteness and manager status for raked weighting

Additional file 1: Figure S1A shows the patterns for males, sorted by the two Euclidean distances, such that asthmagen groups with similar profiles of prevalence of exposure across all occupational groups are neighbours. In Additional file 1: Figure S1B, the patterns for females are shown, in the same order as for the males. In men, the occupational groups with highest prevalence of exposures are farming, food preparation, wood work, painting/printing and carers. In women, they are farming, food preparation, wood work, hairdressers, and cleaning. In men, bioaerosols, metal, arthropods, latex and aldehydes are the most common asthmagen groups, and in women they are latex, cleaning agents, arthropods, bioaerosols and ammonia.

In the lower left corner occupational and asthmagen groups with the lowest prevalence are clustered together. In men the occupational groups with low prevalences are security/safety, retail, transport, metal workers, and managers. In women they are education, manufacturing, mining, retail, transport, and manager. The asthmagen groups with the lowest prevalence in men were medicinal drugs, reactive dyes, ethylene oxide, flowers and pesticides and in women they were drugs, reactive dyes, epoxy, anhydrides and solder flux.

The Additional file 1 also shows the agents to which workers within each occupational group are simultaneously exposed, e.g. male metal workers are not only exposed to metal, but also to bioaerosols, aldehydes, acrylates, epoxy and anhydrides.

With regard to agents, the large molecular weight organic agents such as bioaerosols and arthropods and mites were more prevalent than exposure to smaller-molecular weight agents although exposure to metals was common among men in occupational groups such as farming, painting/printing, electrical, metal and mechanical workers (Additional file 1).

The most common exposure in our study was bioaerosols (29 % males, 16 % females). This group included moulds such as *Alternaria, Chrysonilia sitophilia, Neurospora* and *Penicillium*, where exposure was likely to have occurred mainly when people were in contact with rotting foodstuffs. In addition, cutting oils potentially contaminated with bacteria were included in this group. Exposure to bioaerosols was common in a wide range of jobs including farming and gardening, food preparation and service, cleaning, carers, and metal/electronics work.

Similar occupational groups were exposed to the arthropods/mites group which included all types of mite as well as caddisflies, crickets and locusts, flour moths, fruit flies, mealworms, sheep blowflies, and silkworms. This exposure was found for 25 % of males and 16 % of females.

Metal exposure included exposure to the metal or compounds of aluminium, chromium, cobalt, nickel, platinum, rhodium, titanium, tin, tungsten carbide, vanadium, stainless steel, or zinc oxide, as well as gas metal arc welding on uncoated mild steel and welding fumes. About 26 % of males and 6 % females, mainly tradespeople, were exposed to one or more of these metals.

## Discussion

This study provides much-needed information about the prevalence of exposure to asthmagens in Australian workplaces. These results will have significant implications for the prevention of occupational asthma, as they provide an important input to the determination of where to focus regulatory activities and inform strategies for risk reduction.

We found that about 47 % of males and 40 % of females were currently likely to be exposed to one or more asthmagens in their jobs. A multinational study found that 26 % of subjects reported occupational exposure in their current job to “vapors, gas, dust or fumes” [8]. Our study investigated a wide range of exposures that were not included in this multinational study (such as latex, arthropods and mites, animal products, and various foods) so this may account for our prevalence being higher. In a Norwegian cohort, a job exposure matrix was applied to all jobs held for the previous 10 years to estimate exposure to biological dust, mineral dust and gas or fumes [9]. Exposure to any of the agents was seen in 62.4 % of men and 58.6 % of women at some time during the past 10 years. Job exposure matrices assign all people in the same job the same exposure [24] whereas our study was able to differentiate between workers in the same job who carried out different tasks.

Latex exposure was one of the few exposures found as commonly amongst females (25 %) as males (22 %). Latex exposure was assigned to respondents who reported wearing latex gloves, regardless of whether the gloves were powdered or not. Latex exposure is known to cause asthma, and efforts have been made to reduce exposure, including introducing powder-free latex gloves or replacement with nitrile gloves [25]. Exposure still seems relatively common in Australia, although it is unknown how well workers were able to differentiate the different types of glove.

Registries of occupational asthma have found common agents to be: isocyanates and latex in South Africa [26]; moulds, animal epithelia and flour, grain and grain mites in Finland [27]; wood dust in Australia [28]; and isocyanates, metal working fluids, adhesives, chrome, latex and glutaraldehyde in the United Kingdom [29]. Physician reporting of occupational asthma to voluntary registries is known to be an under-representation of the total number of cases, and may be biased by diagnosis being related to the presence of a commonly recognized or deemed cause [28]. A South Korean study which collected data from a range of sources (including physicians, surveillance systems and compensation schemes) found the most common agents to be isocyanates, flour/grain, metal, reactive dyes, and solvents [30].

We used an automated version of the expert assessment method to obtain our estimates of exposure. It is not practical to monitor exposure in individual workplaces on a national level to estimate prevalence of exposure. Other estimates have been based on self-reported exposures but have been shown to result in reporting bias [8] and there are concerns that workers do not always know the specific components of materials with which they work. Job Exposure Matrices have also been used, but these result in all the workers in a particular job being allocated the same exposure, ignoring inter-individual variability, potentially resulting in an overestimation of the exposure prevalence. The expert assessment method used in this study [31] differentiates between people in the same job by asking respondents about tasks and processes. Experts then review the answers and assign exposures. We used OccIDEAS, which is based on this expert method, but automates the exposure assessment by using algorithms to assign the same exposure to the same combination of answers without the need for manual review of every case. This method provides individualized and consistent exposure assessment of all respondents based on their self-reported occupational tasks.

### Limitations

We attempted to obtain a random sample of the population for this study. The distribution of our sample was reasonably similar to the Australian labour force, and we weighted by age group and remoteness when extrapolating our numbers. However, we only discovered the erroneous method of “random sampling” used by the commercial company after the data collection was complete. There is no way to know what bias arises from our sample over-representing people with last names beginning with L and M only. Some ethnicities may be under- or over-represented (e.g. Lee/Li or names with the prefix Mac or Mc) but this is difficult to quantify. Additional exploration of the data collected on country of birth found no differences of note between our sample and the Australian working population. Further, there is no reason to think that people with particular initials are preferentially selected into particular occupations, so we think it unlikely to bias the results.

We had no response to our phone call for about a quarter of the sample of phone numbers. We attempted to contact each number 10 times, and phone calls were made at different times of the day and on weekdays as well as weekends. However, it is likely that there was some selection bias with regard to particular occupations, particularly those who work away from home for extended periods such as long-haul truck drivers, and fly-in fly-out mine workers.

We decided to develop 27 groups for the 277 asthmagens [11] and based our groups broadly on the ones used in a previous job exposure matrix [32]. This meant, for example, that exposure to the group “Derived from animals” could have meant the person was exposed to any or all of 13 agents in the group including bat guano, casein, mice, frogs or cattle. We felt this was preferable to assessing each of the 277 asthmagens separately or concentrating only on a small number of specific agents. Further analyses of these data could examine exposure to one or more of the groups in more detail, including which of the specific agents were most common.

## Conclusions

Occupational asthma is an ideal candidate for prevention and these results present clear opportunities for policy action which would be of practical benefit. We contend that these decisions should be based on evidence as to which agents are most commonly encountered in workplaces and which workers are most likely to be exposed to one or more asthmagens (e.g. farmers) which have not previously been available. Our study has provided some of this evidence and further analysis will show whether available controls are being used.

For pulmonary medicine specialists, our study provides an overall picture of which asthmagens are found in which occupations. While the pattern of use of some agents, such as isocyanates and latex, are well understood, our study provides a wider range range of possible causes for physicians to consider in their consultations with patients.

### Ethics approval and consent to participate

This study was approved by the Curtin University Human Research Ethics Committee.

## Availability of data and materials

The datasets supporting the conclusions of this article are available upon request to the corresponding author.

## Additional file

Additional file 1: Figure S1. Occupational groups by asthmagen groups for (A) men, and (B) women. Groups are sorted by decreasing Euclidean distances in men as a measure of similar exposure and displayed for women using the same order of occupational groups and asthmagen groups as for men. The size of the dots represents the prevalence of exposure in each cell. (EtOH - Ethylene oxide, d – derived, Reactives – Other reactive chemicals, Cleaning – Industrial cleaning and sterilizing agents, Arthropods – Arthropods and Mites). (PDF 135 kb)

## Abbreviations

ANZSCO: Australian and New Zealand Standard Classification of Occupations
ARIA+: Accessibility/remoteness index of Australia
AWES: Australian Workplace Exposures Study
CI: confidence interval
JSM: job specific module
OR: odds ratio
SEIFA: socio-economic indexes for areas disadvantage score
