# Supplementary figures and images for: The estimated prevalence of exposure to asthmagens in the Australian workforce, 2014
Source: BMC Pulm Med. 2016 Apr 9;16:48. doi: 10.1186/s12890-016-0212-6 (PMC4826519; doi:10.1186/s12890-016-0212-6)

## A Men

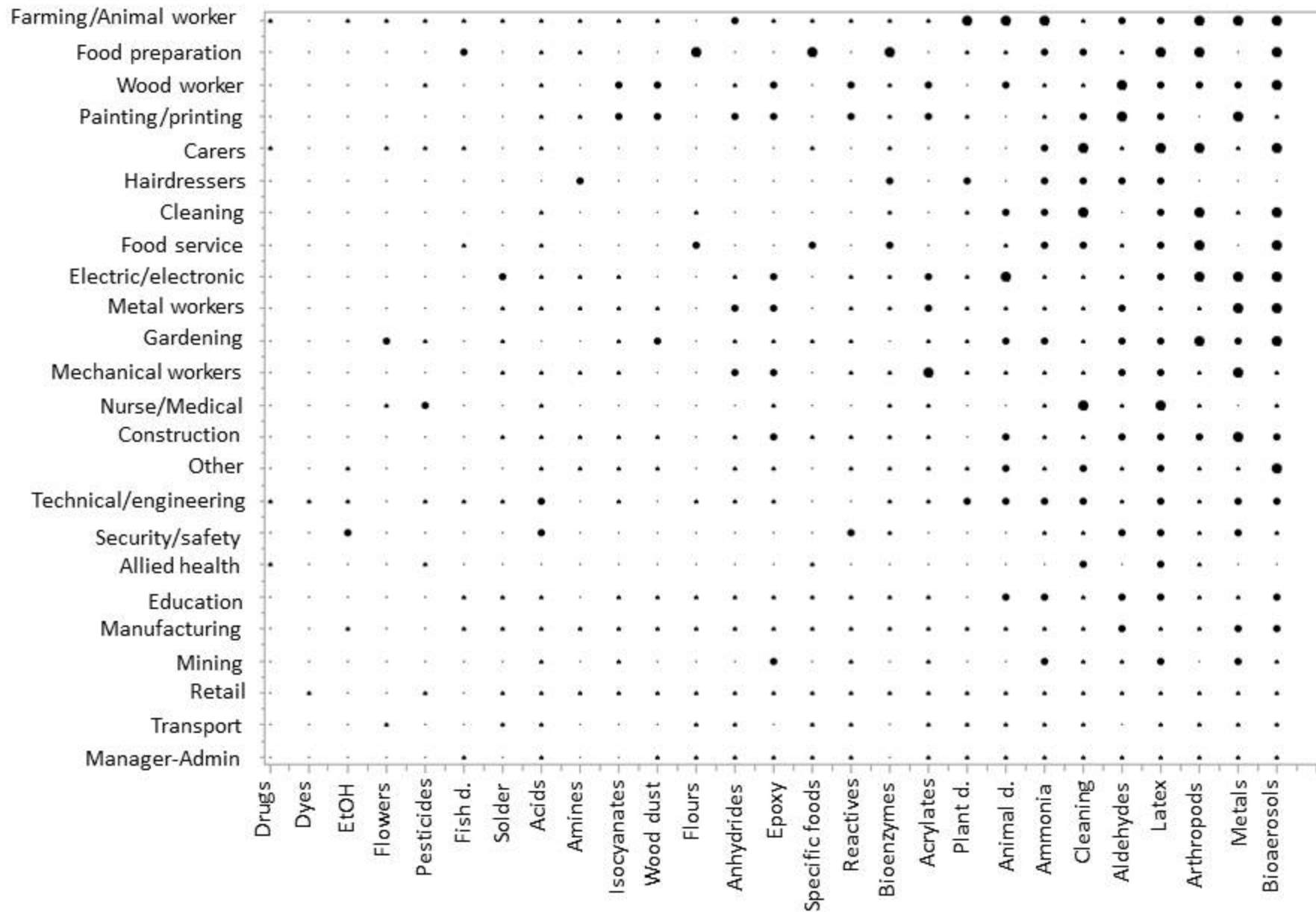

### B Women

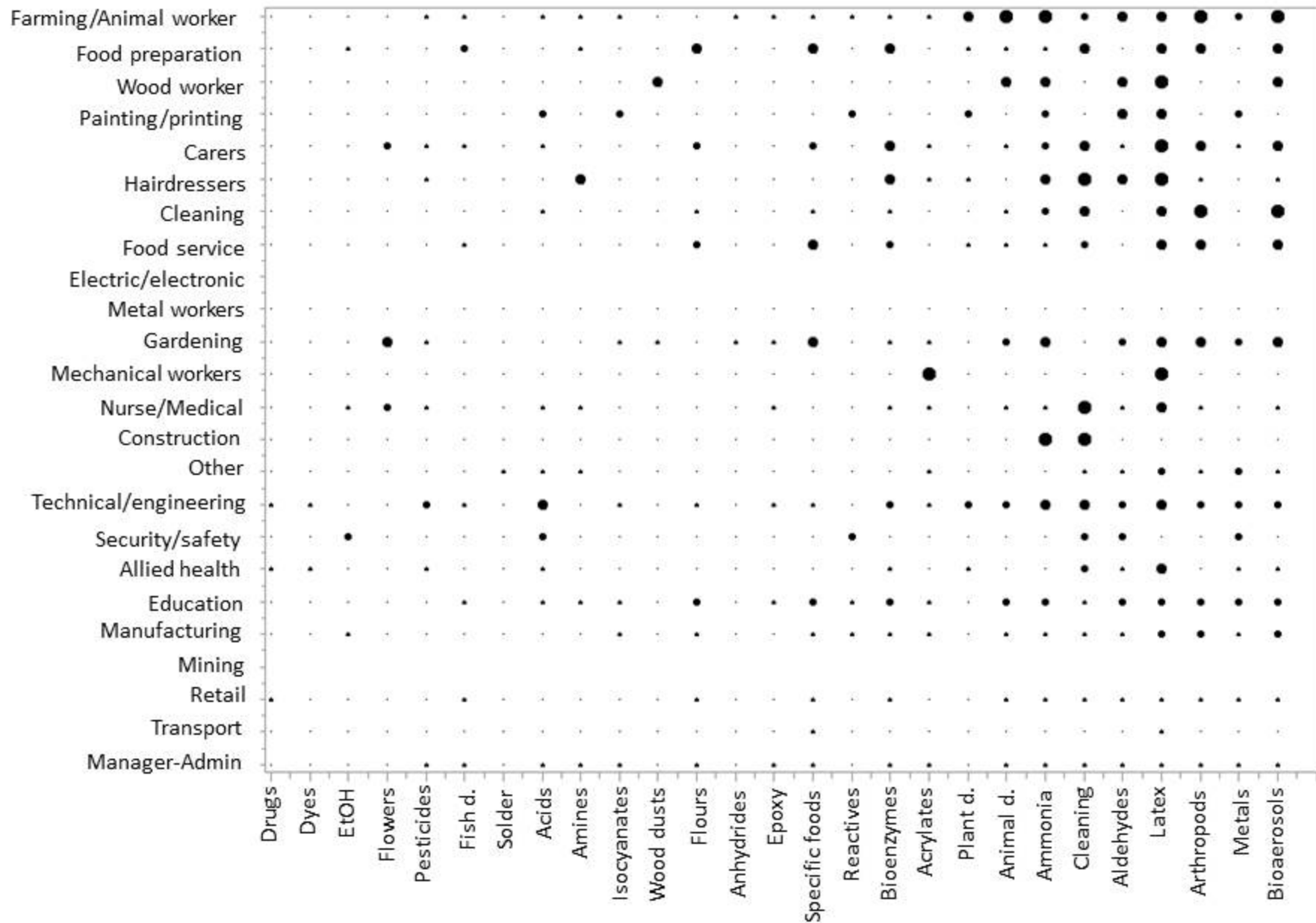

Supplement: Additional file 1: Figure S1. — Occupational groups by asthmagen groups for (A) men, and (B) women. Groups are sorted by decreasing Euclidean distances in men as a measure of similar exposure and displayed for women using the same order of occupational groups and asthmagen groups as for men. The size of the dots represents the prevalence of exposure in each cell. (EtOH - Ethylene oxide, d – derived, Reactives – Other reactive chemicals, Cleaning – Industrial cleaning and sterilizing agents, Arthropods – Arthropods and Mites). (PDF 135 kb) [file 12890_2016_212_MOESM1_ESM.pdf]
